# The Role of the Microbiota in the Pathogenesis and Treatment of Atopic Dermatitis—A Literature Review

**DOI:** 10.3390/ijms25126539

**Published:** 2024-06-13

**Authors:** Martyna Wrześniewska, Julia Wołoszczak, Gabriela Świrkosz, Hubert Szyller, Krzysztof Gomułka

**Affiliations:** 1 Student Scientific Group of Internal Medicine and Allergology, Faculty of Medicine, Wroclaw Medical University, 50-368 Wroclaw, Poland; martyna.wrzesniewska@student.umw.edu.pl (M.W.); julia.woloszczak@student.umw.edu.pl (J.W.); gabriela.swirkosz@student.umw.edu.pl (G.Ś.); hubert.szyller@student.umw.edu.pl (H.S.); 2 Clinical Department of Internal Medicine, Pneumology and Allergology, Faculty of Medicine, Wroclaw Medical University, 50-368 Wroclaw, Poland

**Keywords:** atopic dermatitis, eczema, gut microbiome, microbiota, probiotics, diet, hygiene, treatment, environment, atopy

## Abstract

Atopic dermatitis (AD) is a chronic inflammatory skin condition with a high prevalence worldwide. AD pathogenesis is complex and consists of immune system dysregulation and impaired skin barrier, influenced by genetic and environmental factors. The purpose of the review is to show the complex interplay between atopic dermatitis and the microbiota. Human microbiota plays an important role in AD pathogenesis and the course of the disease. Dysbiosis is an important factor contributing to the development of atopic diseases, including atopic dermatitis. The gut microbiota can influence the composition of the skin microbiota, strengthening the skin barrier and regulating the immune response via the involvement of bacterial metabolites, particularly short-chain fatty acids, in signaling pathways of the gut–skin axis. AD can be modulated by antibiotic intake, dietary adjustments, hygiene, and living conditions. One of the promising strategies for modulating the course of AD is probiotics. This review offers a summary of how the microbiota influences the development and treatment of AD, highlighting aspects that warrant additional investigation.

## 1. Introduction

Atopic dermatitis (AD), also known as atopic eczema or eczema, is one of the most prevalent chronic inflammatory skin diseases, affecting approximately 10% of adults and 20% of children, being one of the most common childhood skin disorders, with its prevalence still increasing globally over the last few decades [1,2]. AD has age-dependent symptoms that often co-occur with other atopic, IgE-associated diseases (food allergies, allergic rhinitis, or allergic asthma). Interestingly, AD frequently marks the onset of the “atopic march”, a progressive sequence of allergic conditions that emerge during early childhood, which commonly leads to the development of asthma or rhinitis in a significant portion of affected individuals. The most common manifestations of the disease include intense itching, dry skin, recurrent eczematous lesions, and lichenification. It can appear at any age, but the most typical time of the disease onset is 3 to 6 months [1,2,3,4].

Common eczema locations in infants are the scalp, face, neck, trunk, and the outer parts of the arms and legs, with the diaper area typically unaffected. In infants, Yamamoto’s sign can be seen—the midline of the face and the tip of the nose are always spared [5]. Children tend to experience eczema in the creases of their arms and legs, neck, wrists, and ankles. As individuals enter adolescence and adulthood, eczema tends to affect the flexural surfaces of the arms and legs, as well as the hands and feet. Itchiness, a hallmark of eczema, persists throughout the day and often intensifies at night, resulting in sleep disturbances and significant declines in quality of life regardless of age [6,7]. AD diagnosis is generally based on Hanifin and Rajka’s diagnostic criteria [8].

AD pathogenesis is complex and includes genetic and environmental factors, impaired skin barrier, skin microbiota alterations.

### 1.1. Genetic Factors

One of the common genetic alterations causing eczema is gene encoding filaggrin (FLG) mutation [9]. Filaggrin is an epidermal structural protein in the stratum corneum responsible for maintaining the skin barrier. Its breakdown products, urocanic acid and pyrrolidine carboxylic acid, are responsible for epidermis moisturization and the skin’s acidic pH [10]. FLG mutation results in loss of protein function and, consequently, damage to the epidermis; homozygous mutations are linked to a higher likelihood of experiencing severe AD characterized by earlier onset, prolonged duration, and susceptibility to skin infections [3,4,9]. Studies showed that FLG-deficient mice demonstrate a reduced stratum corneum barrier function with enhanced sensitization and can develop spontaneous dermatitis on proallergic BALB/c background [11,12]. Filaggrin mutation is detected in up to 30% of AD patients, and it also increases the patient’s risk of developing other skin conditions, such as ichthyosis vulgaris or keratosis pilaris. Claudins, proteins crucial for forming tight junctions in the skin, airways, and GI tract epithelium, were found to be associated with the risk of allergic sensitization [13,14]. Polymorphisms in the CLDN-1 gene encoding claudin-1 were found to be associated with AD, and downregulation of claudin-1, claudin-4, and claudin-23 was reported in the skin of AD patients [14,15,16]. Further factors contributing to epidermis defect are a decreased level of ceramides in the stratum corneum, resulting in transepidermal water loss [4]. In AD patients, the level of ceramides with an extremely short chain length is significantly increased, and the number of long-chain ceramides decreases; compared to healthy subjects, patients with AD have an increase in ceramide subclasses AS, AH, AP, ADS, and NS and a decrease in subclasses NP, NH, and acyl-CERs [17]. In a study by Angelowa-Fischer et al., reduced ceramide/cholesterol ratios in AD skin have been reported [18]. Changes in stratum corneum lipid content correlate with disease severity but are independent of filaggrin mutations [19].

### 1.2. Immune Factors

The immune onset of AD originates from the infiltration of allergens entering the compromised epidermal barrier and engaging IgE on inflammatory dendritic epidermal cells (IDECs) and epidermal Langerhans cells (LCs) [20]. IgE-activated IDECs and LCs then release pro-inflammatory cytokines such as thymic stromal lymphopoietin (TSLP), CCL17, CCL18, CCL22, and IL-33 [20]. This initiates a sensitization cascade mediated by T cells and, consequently, a cutaneous inflammation. The Th2 immune response triggers the production of IL-4, IL-13, IL-31, and IL-22 and further compromises the skin’s barrier function by diminishing the expression of epithelial barrier molecules—FLG, lorcrin, PPL, and claudins [21,22]. Moreover, they cause pruritus by direct stimulation of sensory neurons [23]. Scratching in response to pruritus exacerbates the activation and infiltration of pro-inflammatory cells, which release chemokines like CCL17 and TSLP, intensifying the inflammatory cascade [20,24,25,26]. Innate lymphoid cells (ILCs) play a major role in shaping immunity, tissue homeostasis, and inflammation. Changes in ILCs, especially ILC2s, contribute to the onset and progression of AD, and ILC2s are connected to epithelial damage [27,28]. In AD patients, ILC2 cells are elevated in the skin affected by the disease, where they show an activated phenotype [29,30]. Expansion of ICL2 cells is associated with elevated type-2 cytokines production and inflammatory skin lesions [31,32]. On the other hand, a natural killer (NK) cell blood deficiency is presumed to be involved in inflammation associated with AD [33].

### 1.3. Environmental Factors

Environmental factors play an important role in AD development and include climate changes, air pollutants, irritants, water hardness, urban living, diet, and multiple other factors [34]. Cigarette smoking is also a modifiable risk factor for AD development; active and passive smokers who have higher IgE levels are more likely to develop atopies [35,36,37]. Climate change can explain AD prevalence between different populations—AD symptoms correlate negatively with annual outside temperature and positively with latitude [38,39,40]. UV light has an immunosuppressive effect, partly because it participates in the transformation of trans-urocanic acid into cis-urocanic acid within the skin filaggrin, which also has immunosuppressive properties [41]. Exposure to sunlight/UVB increases vitamin D serum levels, which has a protective effect on AD exacerbation [42]. Air pollutants are associated with the development and aggravation of AD [43]; their detrimental effect is connected to the production of reactive oxygen species, leading to damage of proteins, lipids, and DNA of the stratum corneum [44]. In animal experiments, oxidative stress in the skin elicits itching and scratching, with the infiltration of inflammatory cells and increased expression of IL-4 [45].

### 1.4. Microbiota

Skin serves as the primary barrier between the human body and the outside world, and its microbial diversity is impacted by the diversity of environments we inhabit. The microorganisms in symbiosis with the host are crucial for the development and maintenance of healthy keratinocytes and the epidermis. The microbial communities significantly contribute to homeostasis and regulating immune function. Microbiota alterations result in broader implications for overall health. There is growing evidence that dysbiosis of the gut microbiota is associated with the pathogenesis of both intestinal and extra-intestinal disorders [46]. Therefore, any factors that disrupt the balance and health of the microbiota can predispose to multiple skin diseases as well as other inflammatory and autoimmune disorders [47,48,49].

In this review, we aim to consolidate recent research findings concerning the human microbiota involvement in AD development and therapeutic approaches. Additionally, we aim to accentuate key areas necessitating further investigation in this field.

## 2. Human Microbiota and Atopic Dermatitis

The skin is the largest organ of the human body and, at the same time, is the most exposed to external factors with which the body comes into contact [50]. Not only does it constitute a physiological barrier, but it is also populated by a variety of bacterial strains, particularly from the *Actinobacteria*, *Firmicutes*, *Proteobacteria*, and *Bacteriodetes* families, especially *Staphylococcus*, *Corynebacterium*, and *Propionibacterium* [51]. With properly functioning mechanisms that keep human skin healthy, commensal human skin bacteria promote immune function in several diverse ways. First, they occupy an ecological niche that could be occupied by pathogenic species in the absence of a physiological microbiota [52]. Some strains also fight pathogens actively: *Staphylococcus epidermidis* produces antibiotics, which exhibit antibacterial properties and fight skin colonization by *Staphylococcus aureus*. Moreover, *S. epidermidis* promotes skin wound healing and prevents tumors by counteracting Toll-like receptors (TLR) 3 signal on keratynocytes, thus reducing inflammation induced by injury [52,53,54]. *Cuticabacterium acnes* converts skin triglycerides found on the skin into short-chain fatty acids (SCFAs) via fermentation, which maintain an acidic pH of the skin surface, inhospitable to many other bacteria from the external environment, and exhibits immunomodulatory properties [48,52].

However, everything changes when dysbiosis occurs, a state of disruption of the quantitative and qualitative composition of the skin microbiota due to negative internal or external factors. One of the skin diseases that has been hypothesized to be associated with the occurrence of dysbiosis is AD.

AD is associated with an increase in the abundance of *S. aureus* in the skin [55,56]. It has been shown that altered skin in AD patients is colonized by *S. aureus* in 70% of cases, while unaltered skin is colonized by *S. aureus* in 39%; the rate is also high when nostrils are sampled—62% [56]. *S. aureus* can colonize 60–100% of the skin surface of an AD patient, while this proportion is 5–30% in healthy individuals [57]. It should be mentioned that the proportion of Methicillin-resistant *S. aureus* (MRSA) is increasing among patients suffering from AD. The reservoir of the bacteria is not only the nostrils but also the affected skin itself, resulting in permanent recolonization, recurrent skin infections of the same etiology, and, consequently, persistent chronic inflammation [56,57,58]. Treatment and eradication from the skin surface of over-represented *S. aureus* is further complicated by a dense biofilm that is resistant to antibiotic therapy [51]. Moreover, interruptions in the skin barrier caused by scratching allow *S. aureus* to colonize and proliferate within the underlying layers [59].

An overrepresented population of *S. aureus* contributes in several ways to the disruption of the natural microbiota and the unsealing of the skin barrier. At the colonization stage, *S. aureus* binds to the stratum corneum, facilitated by its pathological deformation and the presence of fibronectin. It then secretes virulent factors, among which are proteolytic enzymes, as well as stimulates the production of endogenous keratinocyte proteases. Thus, the skin barrier is breached and, as a consequence, other bacterial virulence factors stimulate a type II immune response, stimulating mainly Th2 and Th17 lymphocytes to overproduce the cytokines IL-1, IL-12, IL-4 and IL-22, successively disrupting the modulation of the immune system response, *S. aureus* superantigen also causes degranulation of mast cells and basophils and, as a consequence, histamine secretion and overproduction of IgE antibodies [50,57,60]. It also contributes to raising the pH of the skin and altering the lipid profile, thereby reducing the level of AMP produced against *S. aureus* by commensal skin bacteria [57,61].

Importantly, dysbiosis involving an overgrowth of *S. aureus* is not the only possible cause of increasing AD symptoms. More recently, an overgrowth of *S. epidermidis* has also been suspected as a potential driver of inflammation due to the overproduction of phenol-soluble modulin (PSM), which induces skin inflammation similar to *S. aureus* [53,54].

A similar effect is suspected in the case of an overabundant population of *Malassezia furfur*, which belongs to fungi and is also present on the skin like a commensal element of the microbiota. Features typical of AD, such as decreased B-defensins, alkaline pH, and irritated skin surface, may favor colonization of *M. furfur* [62]. It is suspected to be a helpful marker for assessing the development of AD, based on IgE antibody count growth due to hypersensitivity to *M. furfur*, especially when it comes to hand and neck dermatitis (HND)—one of the phenotypes of AD [34,62].

## 3. Gut Microbiota in Atopic Dermatitis

In recent years, theories have been emerging linking the occurrence and exacerbation of AD to not only the skin microbiota but also the gut microbiota, which is the commensal flora of our digestive system [63]. Changes in the composition of the gut microbiota have previously been linked to allergy and asthma symptoms [64,65], among others.

The reciprocal influence of gut and skin microbiota, the so-called “gut–skin axis”, has been proposed to play a role also in the development and symptoms of AD. The gut microbiota is the largest endocrine organ, being the source of number of hormone-like metabolites and signal molecules [43,44,45]. Its high diversity is a protective factor regulating immune responses in the host [46,47]. The formation of the intestinal microbiota is a dynamic process, ending more or less at the stage of the final termination of breastfeeding and the transition to solid food around 2–3 years of age when the body’s microbiota adopts the composition it will retain under physiological conditions until adulthood [66]. Years of co-evolution between humans and commensal bacteria have made the immune system able to distinguish them from pathogens. Therefore, it is recommended to promote the development of the physiological intestinal flora from childhood through min. breastfeeding, the use of probiotics with the aim of preventing the development of atopy [67,68,69]. In AD patients, there is a significant depletion of the intestinal microbiota, ex. *Lactobacillus* and *Bifidobacterium*, with a concomitant overrepresentation of *Escherichia coli* and *Clostridium difficile* and, importantly, as in the case of the skin microbiota, an abundant occurrence of *S. aureus* [68,69,70]. The mycobiota, the fungal part of the microbiota, is also altered in atopy: there is a reduced occurrence of *Malassezia* with *Saccharomycetales*, *Rhodotorula*, and *Candida* increase [71,72,73].

**Figure 1:**
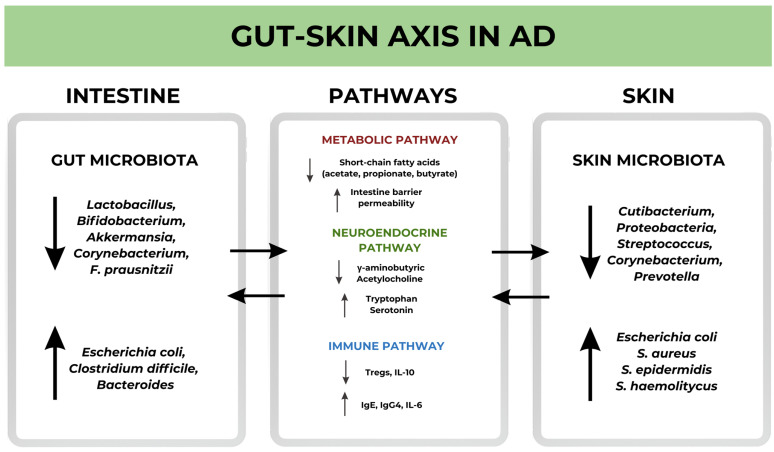
Gut–Skin axis in AD. Changes in the composition of gut and skin microbiota related to interacting with each other through signaling pathways. Decreased (↓) activity of intestinal commensals: *Lactobacillus*, *Bifidobaterium*, *Akkermansia*, *Corynebacterium* and *F.prausnitzii*, and an increase (↑) in the activity of other intestinal residents: *E.coli*, *Clostridium difficile* and *Bacteroides*, causes a disruption in the transmission of signaling pathways which affects the structure of the skin microbiota.

Skin and gut interact not only with each other but also with the brain, forming the so-called gut–brain–skin axis in which the three organs communicate via several pathways (Figure 1) [72,73].

### 3.1. Metabolic Pathway

Gut microbiota modulates homeostasis of the innate and adaptive immune system via its metabolites such as SCFAs, amino acids, vitamins, and bile acid metabolism products. Among them, the most common SCFAs, butyrate, propionate, and acetate, are products of fiber fermentation by the gut microbiota that contributes greatly to improving immune response on many levels [69,72,74]. The SCFA quantity depends not only on gut microbiota metabolism but also on individual fibers input and colon absorption.

SCFAs’ main effect is enhancing epithelial barrier function and decreasing its permeability [75,76]. SCFAs, along with other metabolites and signal molecules such as ribosomally synthesized and post-translationally modified peptides (RiPPs), amino acid metabolites, oligosaccharides, and glycolipids, can form a mucous layer in the gut [77]. The role of butyrate includes the improvement of tight junctions between intestinal epithelial cells (IECs). Butyrate is particularly responsible for strengthening tight junctions via various pathways. It induces IL-10 receptors, which are crucial for barrier formation, and also regulates the expression of important junctional proteins such as occludin, zonulin, and claudins. High expression of tight junctions limits the interaction of microbes with the lumen and luminal epithelium [78]. Butyrate derived from bacteria affects epithelial O_2_ consumption and leads to the stabilization of hypoxia-inducible factor (HIF), a transcription factor that coordinates barrier protection. Via these actions, butyrate also maintains physiological hypoxia, creating a favorable environment for colon commensals [79]. Furthermore, butyrate has an effect on the epithelial barrier by upregulating tight-junction proteins via the activation of AMP-activated protein kinase [80]. Enhancement of the gut epithelial barrier affects the systemic immune response of an individual by reducing the transport of bacteria, proinflammatory cells, cytokines, and toxins from the intestinal lumen into the bloodstream. Reaching the target tissue, transported components accumulate, and may directly disrupt skin homeostasis, causing damage to tissues [66,73,81].

‘Leaky gut syndrome’ is described in the literature as an inflammation trigger in AD. Indeed, bacterial cells and products that escape from the gut can interact with skin receptors, directly and indirectly affecting the skin or skin’s commensal bacteria [66].

Song et al.’s study describes significant dysbiosis of *F. prausnitzii* species in the fecal samples of AD patients with a concurrent decrease in SCFA [82]. Although several studies implied a correlation between gut and skin microbiota in AD, the mechanisms of direct and indirect interaction remain unclear and require further investigation.

The gut microbiota, via both innate and adaptive immunity processes, improves the skin barrier function and contributes to the repair of damaged skin, as was presented in several studies [83,84]. In clinical trials conducted by Ogawa et al. and Guéniche et al., the oral intake of *Lactobacillus* resulted in a marked decrease in transepidermal water loss, which remains a crucial function of the skin barrier, followed by an increase in circulating TGF-β [85,86]. *L.reuteri* supplementation in mice improved the thickness of the skin, hair growth, and sebocyte production [87].

### 3.2. Immune Pathway

The gut microbiota is instrumental in activating innate and adaptive immune mechanisms that function together to protect the host and regulate intestinal homeostasis. It is important to note that the effects of gut microbiota extend beyond the gut and can significantly impact overall health and well-being. However, the immunological implications of gut microbiota on the development of AD remain largely unknown [88,89,90]. Metabolites of gut commensals such as *Bifidobacterium*, *Lactobacillus*, *Clostridium*, *Bacteroides*, *Streptococcus* play major part in proliferation of B cells as well as differentiation of naïve T cells to other types of Th cells and Tregs, which control inflammation by preventing excessive naïve T cells differentiation, downregulating cellular activities and modulate production of IgE and IgG4 [66,91].

According to a study by Millard et al., butyric acid has a significant impact on the differentiation, maturation, and function of DC and macrophages. It alters the phenotypic differentiation process of DC, as assessed by the persistence of CD14 and decreased CD54, CD86, and HLA class II expression. Cells in the early stage of differentiation treated with butyric acid presented increased phagocytic capability. DC differentiated in the absence of butyrate exhibited decreased anti-inflammatory IL-10 secretion nearly 20-fold decreased compared to monocytes, while the presence of butyrate led to DC that exhibited an intermediate capacity to produce IL-10 [92].

It has been described that increased gut bacterial DNA in the bloodstream of chronic skin conditions patients triggers the inflammatory response [72,93]. For example, segmented filamentous bacteria may stimulate pro-inflammatory Th17 and Th1 cells [94]. Interaction between gut microbiota and Th2 cells in atopies was previously described in the literature. In a study by Fujimura et al. on adult mice exposed to house dust, *Lactobacillus* supplementation led to a reduction in Th2 reactivity to airway allergens [95]. Another study reported the effect of propionate treatment on myelopoiesis, resulting in increased production of macrophages and dendritic cell precursors reaching the lungs and equipped with a high phagocytic capacity but impaired capability to induce Th2 response [96]. The studies mentioned indicate a strong link between the gut microbiota and its metabolites and Th2 cell activity, yet a direct involvement in the pathomechanism of AD requires further investigation.

Gut microbiota and its metabolites impact immune homeostasis via interactions with Toll-like receptors (TLRs) [97]. The superfamily of pattern-recognition receptors is a class of transmembrane non-catalytic proteins that bridge innate and adaptive immunity and can recognize molecules with conserved structures from microorganisms known as pathogen-associated molecular patterns (PAMPs). PAMPs bound by TRLs create complex initiating a signal transduction cascade to activate innate immune responses to eliminate pathogens [98,99,100,101]. A negative correlation between intestinal Enterobacteriaceae and TLR4-induced TNF-a levels was described. Similarly, a reduced abundance of *Ruminococcaceae* in fecal samples of atopic eczema infants has been reported, negatively related to TLR2-induced IL-6 and TNF-a [102]. SCFAs can modulate the production of cytokines and chemokines, as well as gene expression of adhesion molecules, and some SCFAs, especially butyrate, also participate in the activation and apoptosis of immune cells [103,104]. *Bacteroides fragilis*, *F.prausnitzii*, and some *Clostridium* clusters produce metabolites such as retinoic acid and polysaccharide A, which may cause accumulation of Tregs and limphocytes stimulating anti-inflammatory reactions [81,105,106,107].

### 3.3. Neuroendocrine Pathway

The concept of the “gut–brain–skin axis” connects the effect of microbiota modulation and stress-induced systemic and cutaneous inflammation [81]. Microbiota connects the gut–brain axis via direct and indirect pathways. The main agents participating in the axis are norepinephrine, serotonin, acetylcholine, and tryptophan [108]. Examples of direct pathways are tryptophan and γ-aminobutyric produced by the gut microbiota and presenting opposite effects. In AD patients, tryptophan intensifies itching, and γ-aminobutyric suppresses it. Increased serotonin can also trigger itching as a response to inflammation, while decreased acetylcholine levels in lesions of AD may suggest its anti-inflammatory effect [72,75]. Gut microbiota also regulates the concentration of cytokines such as IL-10 and IFN-γ in the bloodstream via its metabolites such as butyrate, resulting in changes in hypothalamic–pituitary–adrenal axis function, followed by anxiety and stress [109,110]. An increase in cortisol levels has the potential to enhance the skin and gut barrier by modulating the levels of circulating neuroendocrine molecules, such as tryptamine, trimethylamine, and serotonin. This modulation may lead to the alteration of intestinal permeability via the activation of cortisol receptors expressed on epithelial, immune, and endocrine cells, along with their local response. Furthermore, it could also influence the composition of the gut microbiota by impacting gut transit time [81,111].

## 4. Microbiota as a Mediator of Atopic Dermatitis

Epidemiological investigations over the years have facilitated the identification of factors possibly influencing the microbiota, such as breastfeeding, hygiene and residency conditions, antibiotics, and diet impacting the development of AD [1,38].

Hygiene and residency conditions are significant factors acknowledged for contributing to AD. Their importance is connected with the hygiene hypothesis—a theory stating that alterations in the environment, particularly the decline in “old friends” (organisms that have co-existed and co-evolved with humans for millennia), could disrupt the development and shaping of the immune system. This disruption may result in inadequate responses to both harmless and harmful stimuli, potentially fostering the development of autoimmune and allergic conditions, including AD [112,113]. Children who live in environments with greater exposure to natural elements are less likely to develop atopic eczema—a phenomenon called the “biodiversity hypothesis” that expands upon the hygiene hypothesis. It suggests that interaction with nature enhances the diversity of the human microbiota and strengthens the immune system [114]. Spending time with farm animals, owning a pet in the family household, or living near species-rich vegetation or land use type has been proven to lower atopy incidences in children in multiple research [25,114,115]. Excessive hygiene procedures exacerbate the course of AD. Washing hands frequently significantly increases the risk of hand eczema and is associated with reduced diversity of skin microbial species—particularly with reduced diversity of skin microbiota and an increase in *Staphylococcus*, a species aggravating AD [116,117]. Sherriff et al. study revealed that in children, increasing levels of hygiene correlate with a higher incidence of wheezing and atopic eczema occurring between the ages of 30 and 42 months, and an association with hygiene score was higher in children with more severe AD [118].

There is some evidence that breastfeeding counteracts the development of AD and modulates the course of the disease. Current research findings indicate that breast milk stands out as the optimal nourishment for infants, as it encompasses not only essential nutrients but also bioactive compounds crucial for facilitating optimal growth and development in early childhood [119]. Delayed gut microbiota maturation during infancy is a characteristic feature observed in pediatric allergic diseases [120]. Breast milk contains various components that can influence crucial aspects of allergy development, including maintaining the integrity of the gut barrier, shaping the composition of the gut microbiota, and promoting the development of oral tolerance [121]. During breastfeeding, among other substances, the infant receives beneficial bacteria, including *Lactobacillus*, *Bifidobacterium*, *Streptococcus*, *Staphylococcus*, and *Enterococcus* species [119,122]. Breastfeeding might mitigate the adverse effects of illnesses on an infant’s gut microbiota, such as reducing the likelihood of dysbiosis triggered by conditions like diarrhea and results in allergic children having fecal microbiota more similar to those of healthy individuals compared to formula-fed infants [123]. The third most prevalent solid constituents in breast milk are human milk oligosaccharides (HMOs). They serve as prebiotic agents—indigestible components, promoting the growth of beneficial microorganisms, particularly *Bifidobacterium*, which prevails in the gut of breastfed infants [124,125]. They significantly impact the microbiota in the infant’s gut, thus influencing the maturation of the immune system. In general, breastfed infants have a lower risk of developing autoimmune diseases [126]. Breast milk contains α-tocopherol, β-tocopherol, and prolactin, which reduce infant sensitivity and enhance immune system activity [127]. Breast milk contains TGFβ, an important cytokine that plays a crucial role in suppressing both the Th1 and Th2 pathways in the immune system [128]. Research suggests that the presence of TGFβ-1 or TGFβ-2 in breast milk is associated with a reduced risk of atopic conditions during infancy and early childhood [129]. A study by Morita et al. showed that a lower concentration of TGFβ-1 in human milk at 1 month, but not TGFβ-2, may be correlated with the development of AD in infants [130]. Postbiotic substances (metabolites of intestinal bacteria) derived from *Lactobacillus* spp. may exhibit immunomodulatory effects by diminishing levels of Th2-associated cytokines [131]. KOALA birth cohort study showed that AD could be prevented by breastfeeding in the first 2 years of life in children without first-order relatives with atopy [132]. More recent studies showed that breastfeeding for three or four months (and more) reduces the risk of developing AD [133]; it can also delay the onset of eczema in children [134]. On the other hand, other studies deny the impact of breastfeeding on AD, which is why it still remains a subject of debate [135,136].

Antibiotics have been proven to impact the microbiota and the course of AD. In a study by Watanabe J. et al., conducted on NC/Nga mice with AD-like skin lesions, oral administration of kanamycin resulted in decrease of beneficial *Lactobacillus*, higher total IgE levels, induction of TH2-modulated immune responses, and higher scratching frequency. Another study showed that administering azithromycin in mice with AD-like symptoms resulted in enhanced severity of lesions, promotion of inflammatory cell skin infiltration, elevated levels of IL-4, IL-6, and IL-17A, alongside increased serum TNF-α and IL-6. Furthermore, an increase in gut bacterial genera (*Bacteroides*, *Saccharibacteria*, *Acetatifactor*, *Firmicutes*) and a decrease in three SCFA-producing gut bacterial genera (*Alistipes*, *Clostridiales*, *Butyricicoccus*) was noted [137]. Antibiotic administration disrupts the mother’s own microbiota, which plays a crucial role in fetal development. This maternal imbalance caused by antibiotic exposure can be passed on to her children, affecting the infant’s gut microbiota. Over time, this may impact the health of the children in their later years [138,139]. There is a higher likelihood of childhood AD observed in cases where mothers used antibiotics during pregnancy [140]. In utero exposure to antibiotics, regardless of trimester, has been proven to raise the risk of eczema by 38%, with a more pronounced effect noted in children exposed to penicillin [141,142]. Yamamoto-Hanada et al. discovered that the notable connection to eczema was primarily influenced by the use of macrolides [143]. Studies have shown positive associations between exposure to antibiotics and AD, especially within the first year of life [136,142,143]. Postnatal antibiotic exposure in infants is also associated with an increased risk (40–80%) of developing atopic eczema [122,141]. In a study by Li Y et al., in a group of children under 7 years old, frequent antibiotic use (intravenously or orally) strongly correlated with AD in young adulthood [144]. Antibiotics should be administered thoroughly in the prenatal and early postnatal life; opting for narrow-spectrum antibiotics is advisable because they have a more limited impact on the microbiota, considering the link between prenatal antibiotic use and a higher risk of developing AD, alongside other diseases [138] (Table 1).

## 5. Probiotic Intervention as a Novel Direction in the Treatment of Atopic Dermatitis

The onset of gastrointestinal disorders frequently coincides with the appearance of skin lesions, hinting at a reciprocal association between them [145]. Consequently, modifying gut microbiota emerges as a prospective avenue for modulating immune reactivity and enhancing skin conditions in patients afflicted with AD. Although probiotics are recognized for their potential to modulate gut microbiota and improve AD clinical symptoms, their consistent efficacy is still under investigation. Oral administration of *Lactobacillus paracasei strain KBL382* effectively reduced AD manifestations in murine models [146]. This intervention modulated cytokine production in the skin and increased regulatory T cell populations. Concurrently, *KBL382* administration induced substantial reshaping of gut microbiota composition. Woon-Ki et al. observed a significantly increased abundance of *Akkermansia* with administration of *KBL382*. These findings underscore the immunomodulatory and microbiota-altering potential of *KBL382* in mitigating AD course. *L. sakei WIKIM30*, derived from kimchi, was found to enhance the generation of regulatory T cells (Tregs) while reducing Th2-associated cytokines in mice and also to alter the gut microbiota in AD mice—decreasing in *Arthromitus* and *Ralstonia*, and increasing in the *Ruminococcus* levels [147]. The rise in *Ruminococcus* correlated strongly with Treg-related responses, potentially alleviating AD symptoms. These findings indicate that *L. sakei WIKIM30* could modulate allergic Th2 responses, enhance Treg production, and increase beneficial gut bacteria associated with Tregs, suggesting therapeutic promise for AD treatment. The impact of *L. pentosus* supplementation in children with mild to moderate AD was investigated in a randomized controlled trial. Over a 12-week period, children were randomly assigned to receive either *L. pentosus* or a placebo. While both groups demonstrated improvement in AD severity, no notable disparities were observed between them regarding clinical severity, cytokine levels, or gut microbiota composition. Nevertheless, the mean subjective assessments of the Scoring of AD (SCORAD) notably favored the probiotics group over the placebo group [43].

Oral administration of *Lactococcus chungangensis CAU 28(T)* reduced histological signs of atopic skin lesions in mice, including erosion, epidermal and dermal hyperplasia, and inflammatory cell infiltration [148]. It also suppressed the production of various cytokines and chemokines associated with skin inflammation, including IL-4, IL-5, IL-12, IFN-γ, TNF-α, and thymus- and activation-regulated chemokine (TARC), comparable to the effectiveness of tacrolimus, a commonly used topical AD treatment. These results suggest that *L. chungangensis CAU 28(T)* holds promise as a new probiotic option for managing AD symptoms.

The bacteriocins—bifilact Bb-12 produced by *Bifidobacterium lactis Bb-12* and bifilong Bb-46 produced by *B. longum Bb-46* were found to effectively suppress the intestinal growth of *S. aureus* and *E. coli* [149]. These bacteria are recognized as contributing factors to the onset of AD, and their elevated levels are commonly observed in individuals affected by the condition. This suggests a potential therapeutic role for these probiotic strains in managing AD by targeting harmful intestinal bacteria. Enomoto et al. investigated the impact of administering *B. breve M-16V* and *B. longum BB536* to mothers before and after childbirth and to their newborns for six months on the development of allergic diseases in infants. The results demonstrated a significant decrease in the likelihood of infants developing eczema or AD within the first 18 months of life when receiving the probiotics. Analysis of fecal samples revealed alterations in the microbiota composition, including reduced levels of *Proteobacteria* in supplemented mothers during delivery and a positive correlation with infants’ fecal samples at four months. Overall, the findings suggest that prenatal and postnatal *Bifidobacteria* supplementation may effectively prevent allergic disease development [150].

The impact of a probiotic drink containing *L. paracasei Lpc-37*, *L. acidophilus 74-2*, and *B. animalis* subsp. *lactis DGCC 420 (B. lactis 420)* on clinical and immunological factors in both healthy individuals and patients diagnosed with AD was assessed as well. Via a double-blind trial spanning 8 weeks, participants were administered either probiotics or a placebo. Notably, the probiotic strains were detected in fecal samples post-supplementation. While there was a trend towards reduced AD severity in patients, measured by a 15.5% decrease in SCORAD, distinct differences in immune responses were observed between healthy subjects and AD patients following probiotic consumption. Probiotic intake did not influence CD57(+) levels in AD patients, but it was heightened in healthy individuals, and CD4(+) and CD54(+) were reduced effectively in patients, but no change was observed in healthy participants, indicating varying effects on immune modulation [151].

Replenishing beneficial bacteria via probiotic intake not only addresses intestinal dysfunction but also modulates gut microbiota and systemic immune responses [36,146,148]. With prolonged consumption, probiotics hold promise in reshaping the gut microbial environment towards a more balanced composition, which offers avenues for restoring AD [150,152]. Specific strains such as *L. paracasei KBL382* [36], *L. sakei WIKIM30* [147], and *L. chungangensis CAU 28(T)* [149] show potential for alleviating AD symptoms and altering gut microbiota composition. However, further research with large cohorts is needed to clarify their efficacy, optimal dosages, and long-term effects in AD management.

## 6. Conclusions

Gut microbiota is a major contributor of gut–skin axis via signaling pathways consisting of metabolic, neuroendocrine, and immunological components. Its composition induces modulation of the innate and adaptive immune system, permeability of intestinal barrier and activation of stress-related inflammatory mechanisms. Further research of pathomechanism of these interactions allows to locate targets for new therapies and prevention of AD.

The association between gastrointestinal and skin disorders suggests a bidirectional relationship, indicating the potential of gut microbiota modulation to ameliorate skin conditions, especially AD. Experimental studies with probiotics like *Lactobacillus paracasei* strain KBL382 and *L. sakei* WIKIM30 demonstrate their ability to modulate cytokine production, increase regulatory T cell populations, and reshape gut microbiota, offering promise for AD management. Despite promising findings, uncertainties persist regarding the efficacy of certain probiotics in clinical trials, underscoring the need for further investigation to establish their effectiveness and optimal usage in treating AD.

Factors such as hygiene and breastfeeding, residency conditions, antibiotic intake, dietary changes, and use of hydrolyzed formulas are connected with the development of AD. Research suggests that those factors have the potential to impact the microbiota and influence the host immune system, especially in people with a genetic predisposition towards atopic diseases. However, further studies are required to gain a more comprehensive understanding of these relationships.

## Figures and Tables

**Table 1:** Factors mediating the microbiota in the context of atopic dermatitis—a summary.

Factors Mediating the Microbiota	Effect on Atopic Dermatitis
Residency conditions	“Biodiversity hypothesis”—with greater exposure to natural elements, there is a more diverse microbiota, which strengthens the immune system and reduces the risk of developing AD
Excessive hygiene	Excessive hygiene contributes to dysbiosis, with the increased prevalence of Staphylococcus causing higher occurrence and aggravation of AD
Breastfeeding	Breast milk contains: Beneficial bacteria (e.g., Lactobacillus, Bifidobacterium), reducing the likelihood of gut dysbiosis, HMOs in milk working as prebiotics and promoting the growth of beneficial organisms and gut microbiota maturation, α-tocopherol, β-tocopherol, and prolactin reducing infant sensitivity and enhancing immune system activity, Postbiotic substances with immunomodulatory effects which can diminish levels of Th2-associated cytokines, TGFβ, a cytokine that plays a crucial role in suppressing both the Th1 and Th2 pathways in the immune system. Breastfeeding for 3 or 4 months (and more) reduces the risk of AD.
Antibiotics	Antibiotic administration in pregnant women disrupts the mother’s own microbiota, which is crucial for fetal development. There is a higher likelihood of childhood AD after in utero exposure to antibiotics. Postanatal antibiotic exposure is connected with an increased risk of developing AD in children.

## References

[1] Kapur S., Watson W., Carr S. (2018). Atopic Dermatitis. Allergy Asthma Clin. Immunol..

[2] Lugović-Mihić L., Meštrović-Štefekov J., Potočnjak I., Cindrić T., Ilić I., Lovrić I., Skalicki L., Bešlić I., Pondeljak N. (2023). Atopic Dermatitis: Disease Features, Therapeutic Options, and a Multidisciplinary Approach. Life.

[3] Langan S.M., Irvine A.D., Weidinger S. (2020). Atopic Dermatitis. Lancet.

[4] Kolb L., Ferrer-Bruker S.J. (2023). Atopic Dermatitis.

[5] Deleuran M., Vestergaard C. (2014). Clinical Heterogeneity and Differential Diagnosis of Atopic Dermatitis. Br. J. Dermatol..

[6] Weidinger S., Novak N. (2016). Atopic Dermatitis. Lancet.

[7] McKenna S.P., Doward L.C. (2008). Quality of Life of Children with Atopic Dermatitis and Their Families. Curr. Opin. Allergy Clin. Immunol..

[8] Silverberg N.B. (2017). Typical and Atypical Clinical Appearance of Atopic Dermatitis. Clin. Dermatol..

[9] Kim J., Kim B.E., Leung D.Y.M. (2019). Pathophysiology of Atopic Dermatitis: Clinical Implications. Allergy Asthma Proc..

[10] Egawa G., Kabashima K. (2018). Barrier Dysfunction in the Skin Allergy. Allergol. Int..

[11] Kawasaki H., Nagao K., Kubo A., Hata T., Shimizu A., Mizuno H., Yamada T., Amagai M. (2012). Altered Stratum Corneum Barrier and Enhanced Percutaneous Immune Responses in Filaggrin-Null Mice. J. Allergy Clin. Immunol..

[12] Leitch C.S., Natafji E., Yu C., Abdul-Ghaffar S., Madarasingha N., Venables Z.C., Chu R., Fitch P.M., Muinonen-Martin A.J., Campbell L.E. (2016). Filaggrin-Null Mutations Are Associated with Increased Maturation Markers on Langerhans Cells. J. Allergy Clin. Immunol..

[13] Xia Y., Cao H., Zheng J., Chen L. (2022). Claudin-1 Mediated Tight Junction Dysfunction as a Contributor to Atopic March. Front. Immunol..

[14] De Benedetto A., Rafaels N.M., McGirt L.Y., Ivanov A.I., Georas S.N., Cheadle C., Berger A.E., Zhang K., Vidyasagar S., Yoshida T. (2011). Tight Junction Defects in Patients with Atopic Dermatitis. J. Allergy Clin. Immunol..

[15] Gruber R., Börnchen C., Rose K., Daubmann A., Volksdorf T., Wladykowski E., Vidal-Y-Sy S., Peters E.M., Danso M., Bouwstra J.A. (2015). Diverse Regulation of Claudin-1 and Claudin-4 in Atopic Dermatitis. Am. J. Pathol..

[16] Winge M.C.G., Bilcha K.D., Lieden A., Shibeshi D., Sandilands A., Wahlgren C.F., McLean W.H.I., Nordenskjold M., Bradley M. (2011). Novel Filaggrin Mutation but No Other Loss-of-Function Variants Found in Ethiopian Patients with Atopic Dermatitis. Br. J. Dermatol..

[17] Van Smeden J., Bouwstra J.A. (2016). Stratum Corneum Lipids: Their Role for the Skin Barrier Function in Healthy Subjects and Atopic Dermatitis Patients. Curr. Probl. Dermatol..

[18] Angelova-Fischer I., Mannheimer A.C., Hinder A., Ruether A., Franke A., Neubert R.H.H., Fischer T.W., Zillikens D. (2011). Distinct Barrier Integrity Phenotypes in Filaggrin-Related Atopic Eczema Following Sequential Tape Stripping and Lipid Profiling. Exp. Dermatol..

[19] Janssens M., Van Smeden J., Gooris G.S., Bras W., Portale G., Caspers P.J., Vreeken R.J., Hankemeier T., Kezic S., Wolterbeek R. (2012). Increase in Short-Chain Ceramides Correlates with an Altered Lipid Organization and Decreased Barrier Function in Atopic Eczema Patients. J. Lipid Res..

[20] Kader H.A., Azeem M., Jwayed S.A., Al-Shehhi A., Tabassum A., Ayoub M.A., Hetta H.F., Waheed Y., Iratni R., Al-Dhaheri A. (2021). Current Insights into Immunology and Novel Therapeutics of Atopic Dermatitis. Cells.

[21] Santamaria-Babí L.F. (2022). Atopic Dermatitis Pathogenesis: Lessons From Immunology. Dermatol Pr. Concept.

[22] Nakajima S., Nomura T., Common J., Kabashima K. (2019). Insights into Atopic Dermatitis Gained from Genetically Defined Mouse Models. J. Allergy Clin. Immunol..

[23] Oetjen L.K., Mack M.R., Feng J., Whelan T.M., Niu H., Guo C.J., Chen S., Trier A.M., Xu A.Z., Tripathi S.V. (2017). Sensory Neurons Co-Opt Classical Immune Signaling Pathways to Mediate Chronic Itch. Cell.

[24] Eichenfield L.F., Stripling S., Fung S., Cha A., O’Brien A., Schachner L.A. (2022). Recent Developments and Advances in Atopic Dermatitis: A Focus on Epidemiology, Pathophysiology, and Treatment in the Pediatric Setting. Pediatr. Drugs.

[25] Furue M., Chiba T., Tsuji G., Ulzii D., Kido-Nakahara M., Nakahara T., Kadono T. (2017). Atopic Dermatitis: Immune Deviation, Barrier Dysfunction, IgE Autoreactivity and New Therapies. Allergol. Int..

[26] Guttman-Yassky E., Waldman A., Ahluwalia J., Ong P.Y., Eichenfield L.F. (2017). Atopic Dermatitis: Pathogenesis. Semin. Cutan. Med. Surg..

[27] Roediger B., Kyle R., Le Gros G., Weninger W. (2014). Dermal Group 2 Innate Lymphoid Cells in Atopic Dermatitis and Allergy. Curr. Opin. Immunol..

[28] Garofalo C., Cerantonio A., Muscoli C., Mollace V., Viglietto G., De Marco C., Cristiani C.M. (2023). Helper Innate Lymphoid Cells-Unappreciated Players in Melanoma Therapy. Cancers.

[29] Kim B.S., Siracusa M.C., Saenz S.A., Noti M., Monticelli L.A., Sonnenberg G.F., Hepworth M.R., Van Voorhees A.S., Comeau M.R., Artis D. (2013). TSLP Elicits IL-33-Independent Innate Lymphoid Cell Responses to Promote Skin Inflammation. Sci. Transl. Med..

[30] Salimi M., Barlow J.L., Saunders S.P., Xue L., Gutowska-Owsiak D., Wang X., Huang L.C., Johnson D., Scanlon S.T., McKenzie A.N.J. (2013). A Role for IL-25 and IL-33-Driven Type-2 Innate Lymphoid Cells in Atopic Dermatitis. J. Exp. Med..

[31] Roediger B., Kyle R., Yip K.H., Sumaria N., Guy T.V., Kim B.S., Mitchell A.J., Tay S.S., Jain R., Forbes-Blom E. (2013). Cutaneous Immunosurveillance and Regulation of Inflammation by Group 2 Innate Lymphoid Cells. Nat. Immunol..

[32] Imai Y., Yasuda K., Sakaguchi Y., Haneda T., Mizutani H., Yoshimoto T., Nakanishi K., Yamanishi K. (2013). Skin-Specific Expression of IL-33 Activates Group 2 Innate Lymphoid Cells and Elicits Atopic Dermatitis-like Inflammation in Mice. Proc. Natl. Acad. Sci. USA.

[33] Mack M.R., Brestoff J.R., Berrien-Elliott M.M., Trier A.M., Yang T.L.B., McCullen M., Collins P.L., Niu H., Bodet N.D., Wagner J.A. (2020). Blood Natural Killer Cell Deficiency Reveals an Immunotherapy Strategy for Atopic Dermatitis. Sci. Transl. Med..

[34] Glatz M., Bosshard P.P., Hoetzenecker W., Schmid-Grendelmeier P. (2015). The Role of *Malassezia* spp. in Atopic Dermatitis. J. Clin. Med..

[35] Kantor R., Kim A., Thyssen J.P., Silverberg J.I. (2016). Association of Atopic Dermatitis with Smoking: A Systematic Review and Meta-Analysis. J. Am. Acad. Dermatol..

[36] Yi O., Kwon H.J., Kim H., Ha M., Hong S.J., Hong Y.C., Leem J.H., Sakong J., Lee C.G., Kim S.Y. (2012). Effect of Environmental Tobacco Smoke on Atopic Dermatitis among Children in Korea. Environ. Res..

[37] Vork K.L., Broadwin R.L., Blaisdell R.J. (2007). Developing Asthma in Childhood from Exposure to Secondhand Tobacco Smoke: Insights from a Meta-Regression. Environ. Health Perspect..

[38] Bonamonte D., Filoni A., Vestita M., Romita P., Foti C., Angelini G. (2019). The Role of the Environmental Risk Factors in the Pathogenesis and Clinical Outcome of Atopic Dermatitis. BioMed Res. Int..

[39] Weiland S.K., Hüsing A., Strachan D.P., Rzehak P., Pearce N. (2004). Climate and the Prevalence of Symptoms of Asthma, Allergic Rhinitis, and Atopic Eczema in Children. Occup. Environ. Med..

[40] Suárez-Varela M.M., García-Marcos Alvarez L., Kogan M.D., González A.L., Gimeno A.M., Ontoso I.A., Díaz C.G., Pena A.A., Aurrecoechea B.D., Monge R.M.B. (2008). Climate and Prevalence of Atopic Eczema in 6- to 7-Year-Old School Children in Spain. ISAAC PhASE III. Int. J. Biometeorol..

[41] Byremo G., Rød G., Carlsen K.H. (2006). Effect of Climatic Change in Children with Atopic Eczema. Allergy.

[42] Vestita M., Filoni A., Congedo M., Foti C., Bonamonte D. (2015). Vitamin D and Atopic Dermatitis in Childhood. J. Immunol. Res..

[43] Ahn K. (2014). The Role of Air Pollutants in Atopic Dermatitis. J. Allergy Clin. Immunol..

[44] Niwa Y., Sumi H., Kawahira K., Terashima T., Nakamura T., Akamatsu H. (2003). Protein Oxidative Damage in the Stratum Corneum: Evidence for a Link between Environmental Oxidants and the Changing Prevalence and Nature of Atopic Dermatitis in Japan. Br. J. Dermatol..

[45] Saito A., Tanaka H., Usuda H., Shibata T., Higashi S., Yamashita H., Inagaki N., Nagai H. (2011). Characterization of Skin Inflammation Induced by Repeated Exposure of Toluene, Xylene, and Formaldehyde in Mice. Environ. Toxicol..

[46] Garofalo C., Cristiani C.M., Ilari S., Passacatini L.C., Malafoglia V., Viglietto G., Maiuolo J., Oppedisano F., Palma E., Tomino C. (2023). Fibromyalgia and Irritable Bowel Syndrome Interaction: A Possible Role for Gut Microbiota and Gut-Brain Axis. Biomedicines.

[47] Hou K., Wu Z.X., Chen X.Y., Wang J.Q., Zhang D., Xiao C., Zhu D., Koya J.B., Wei L., Li J. (2022). Microbiota in Health and Diseases. Signal Transduct. Target. Ther..

[48] Carding S., Verbeke K., Vipond D.T., Corfe B.M., Owen L.J. (2015). Dysbiosis of the Gut Microbiota in Disease. Microb. Ecol. Health Dis..

[49] Prescott S.L., Larcombe D.L., Logan A.C., West C., Burks W., Caraballo L., Levin M., Van Etten E., Horwitz P., Kozyrskyj A. (2017). The Skin Microbiome: Impact of Modern Environments on Skin Ecology, Barrier Integrity, and Systemic Immune Programming. World Allergy Organ. J..

[50] Byrd A.L., Belkaid Y., Segre J.A. (2018). The Human Skin Microbiome. Nat. Rev. Microbiol..

[51] Hrestak D., Matijašić M., Paljetak H.Č., Drvar D.L., Hadžavdić S.L., Perić M. (2022). Skin Microbiota in Atopic Dermatitis. Int. J. Mol. Sci..

[52] Skowron K., Bauza-kaszewska J., Kraszewska Z., Wiktorczyk-kapischke N., Grudlewska-buda K., Kwiecińska-piróg J., Wałecka-zacharska E., Radtke L., Gospodarek-komkowska E. (2021). Human Skin Microbiome: Impact of Intrinsic and Extrinsic Factors on Skin Microbiota. Microorganisms.

[53] Cau L., Williams M.R., Butcher A.M., Nakatsuji T., Kavanaugh J.S., Cheng J.Y., Shafiq F., Higbee K., Hata T.R., Horswill A.R. (2021). Staphylococcus Epidermidis Protease EcpA Can Be a Deleterious Component of the Skin Microbiome in Atopic Dermatitis. J. Allergy Clin. Immunol..

[54] Williams M.R., Bagood M.D., Enroth T.J., Bunch Z.L., Jiang N., Liu E., Almoughrabie S., Khalil S., Li F., Brinton S. (2023). Staphylococcus Epidermidis Activates Keratinocyte Cytokine Expression and Promotes Skin Inflammation through the Production of Phenol-Soluble Modulins. Cell Rep..

[55] Blicharz L., Rudnicka L., Czuwara J., Waśkiel-Burnat A., Goldust M., Olszewska M., Samochocki Z. (2021). The Influence of Microbiome Dysbiosis and Bacterial Biofilms on Epidermal Barrier Function in Atopic Dermatitis—An Update. Int. J. Mol. Sci..

[56] Bjerre R.D., Holm J.B., Palleja A., Sølberg J., Skov L., Johansen J.D. (2021). Skin Dysbiosis in the Microbiome in Atopic Dermatitis Is Site-Specific and Involves Bacteria, Fungus and Virus. BMC Microbiol..

[57] Kim J., Kim B.E., Ahn K., Leung D.Y.M. (2019). Interactions Between Atopic Dermatitis and Staphylococcus Aureus Infection: Clinical Implications. Allergy Asthma Immunol. Res..

[58] Ogonowska P., Gilaberte Y., Barańska-Rybak W., Nakonieczna J. (2020). Colonization With Staphylococcus Aureus in Atopic Dermatitis Patients: Attempts to Reveal the Unknown. Front. Microbiol..

[59] Blicharz L., Rudnicka L., Samochocki Z. (2019). Staphylococcus Aureus: An Underestimated Factor in the Pathogenesis of Atopic Dermatitis?. Adv. Dermatol. Allergol..

[60] Zhang X.E., Zheng P., Ye S.Z., Ma X., Liu E., Pang Y.B., He Q.Y., Zhang Y.X., Li W.Q., Zeng J.H. (2024). Microbiome: Role in Inflammatory Skin Diseases. J. Inflamm. Res..

[61] Alexander H., Paller A.S., Traidl-Hoffmann C., Beck L.A., De Benedetto A., Dhar S., Girolomoni G., Irvine A.D., Spuls P., Su J. (2020). The Role of Bacterial Skin Infections in Atopic Dermatitis: Expert Statement and Review from the International Eczema Council Skin Infection Group. Br. J. Dermatol..

[62] Navarro-Triviño F.J., Ayén-Rodríguez Á. (2022). Study of Hypersensitivity to Malassezia Furfur in Patients with Atopic Dermatitis with Head and Neck Pattern: Is It Useful as a Biomarker and Therapeutic Indicator in These Patients?. Life.

[63] Liu Y., Du X., Zhai S., Tang X., Liu C., Li W. (2022). Gut Microbiota and Atopic Dermatitis in Children: A Scoping Review. BMC Pediatr..

[64] Melli L.C.F.L., do Carmo-Rodrigues M.S., Araújo-Filho H.B., Solé D., de Morais M.B. (2016). Intestinal Microbiota and Allergic Diseases: A Systematic Review. Allergol. Immunopathol..

[65] Fujimura K.E., Lynch S.V. (2015). Microbiota in Allergy and Asthma and the Emerging Relationship with the Gut Microbiome. Cell Host Microbe.

[66] Kim J.E., Kim H.S. (2019). Microbiome of the Skin and Gut in Atopic Dermatitis (AD): Understanding the Pathophysiology and Finding Novel Management Strategies. J. Clin. Med..

[67] Ogunrinola G.A., Oyewale J.O., Oshamika O.O., Olasehinde G.I. (2020). The Human Microbiome and Its Impacts on Health. Int. J. Microbiol..

[68] Mazur M., Tomczak H., Łodyga M., Plagens-Rotman K., Merks P., Czarnecka-Operacz M. (2023). The Intestinal and Skin Microbiome in Patients with Atopic Dermatitis and Their Influence on the Course of the Disease: A Literature Review. Healthcare.

[69] Fang Z., Li L., Zhang H., Zhao J., Lu W., Chen W. (2021). Gut Microbiota, Probiotics, and Their Interactions in Prevention and Treatment of Atopic Dermatitis: A Review. Front. Immunol..

[70] Alam M.J., Xie L., Yap Y.A., Marques F.Z., Robert R. (2022). Manipulating Microbiota to Treat Atopic Dermatitis: Functions and Therapies. Pathogens.

[71] Glatthardt T., van Tilburg Bernardes E., Arrieta M.C. (2023). The Mycobiome in Atopic Diseases: Inducers and Triggers. J. Allergy Clin. Immunol..

[72] Sinha S., Lin G., Ferenczi K. (2021). The Skin Microbiome and the Gut-Skin Axis. Clin. Dermatol..

[73] Salem I., Ramser A., Isham N., Ghannoum M.A. (2018). The Gut Microbiome as a Major Regulator of the Gut-Skin Axis. Front. Microbiol..

[74] O’Hara A.M., Shanahan F. (2006). The Gut Flora as a Forgotten Organ. EMBO Rep..

[75] De Pessemier B., Grine L., Debaere M., Maes A., Paetzold B., Callewaert C. (2021). Gut–Skin Axis: Current Knowledge of the Interrelationship between Microbial Dysbiosis and Skin Conditions. Microorganisms.

[76] Mariadason J.M., Catto-Smith A., Gibson P.R. (1999). Modulation of Distal Colonic Epithelial Barrier Function by dietary Fibre in Normal Rats. Gut.

[77] Cani P.D., Possemiers S., Van De Wiele T., Guiot Y., Everard A., Rottier O., Geurts L., Naslain D., Neyrinck A., Lambert D.M. (2009). Changes in Gut Microbiota Control Inflammation in Obese Mice through a Mechanism Involving GLP-2-Driven Improvement of Gut Permeability. Gut.

[78] Martin-Gallausiaux C., Marinelli L., Blottière H.M., Larraufie P., Lapaque N. (2021). SCFA: Mechanisms and Functional Importance in the Gut. Proc. Nutr. Soc..

[79] Kelly C.J., Zheng L., Campbell E.L., Saeedi B., Scholz C.C., Bayless A.J., Wilson K.E., Glover L.E., Kominsky D.J., Magnuson A. (2015). Crosstalk between Microbiota-Derived Short-Chain Fatty Acids and Intestinal Epithelial HIF Augments Tissue Barrier Function. Cell Host Microbe.

[80] Pérez-Reytor D., Puebla C., Karahanian E., García K. (2021). Use of Short-Chain Fatty Acids for the Recovery of the Intestinal Epithelial Barrier Affected by Bacterial Toxins. Front. Physiol..

[81] Park D.H., Kim J.W., Park H.J., Hahm D.H. (2021). Comparative Analysis of the Microbiome across the Gut–Skin Axis in Atopic Dermatitis. Int. J. Mol. Sci..

[82] Song H., Yoo Y., Hwang J., Na Y.C., Kim H.S. (2016). Faecalibacterium Prausnitzii Subspecies-Level Dysbiosis in the Human Gut Microbiome Underlying Atopic Dermatitis. J. Allergy Clin. Immunol..

[83] Kim Y.G., Udayanga K.G.S., Totsuka N., Weinberg J.B., Núñez G., Shibuya A. (2014). Gut Dysbiosis Promotes M2 Macrophage Polarization and Allergic Airway Inflammation via Fungi-Induced PGE2. Cell Host Microbe.

[84] Chen Y.H., Wu C.S., Chao Y.H., Lin C.C., Tsai H.Y., Li Y.R., Chen Y.Z., Tsai W.H., Chen Y.K. (2017). Lactobacillus Pentosus GMNL-77 Inhibits Skin Lesions in Imiquimod-Induced Psoriasis-like Mice. J. Food Drug Anal..

[85] Ogawa M., Saiki A., Matsui Y., Tsuchimoto N., Nakakita Y., Takata Y., Nakamura T. (2016). Effects of Oral Intake of Heat-Killed Lactobacillus Brevis SBC8803 (SBL88^TM^) on Dry Skin Conditions: A Randomized, Double-Blind, Placebo-Controlled Study. Exp. Ther. Med..

[86] Gueniche A., Philippe D., Bastien P., Reuteler G., Blum S., Castiel-Higounenc I., Breton L., Benyacoub J. (2014). Randomised Double-Blind Placebo-Controlled Study of the Effect of Lactobacillus Paracasei NCC 2461 on Skin Reactivity. Benef. Microbes.

[87] Levkovich T., Poutahidis T., Smillie C., Varian B.J., Ibrahim Y.M., Lakritz J.R., Alm E.J., Erdman S.E. (2013). Probiotic Bacteria Induce a “Glow of Health”. PLoS ONE.

[88] Cerf-Bensussan N., Gaboriau-Routhiau V. (2010). The Immune System and the Gut Microbiota: Friends or Foes?. Nat. Rev. Immunol..

[89] Garrett W.S., Gordon J.I., Glimcher L.H. (2010). Homeostasis and Inflammation in the Intestine. Cell.

[90] Plaza-Díaz J., Ruiz-Ojeda F.J., Gil-Campos M., Gil A. (2018). Immune-Mediated Mechanisms of Action of Probiotics and Synbiotics in Treating Pediatric Intestinal Diseases. Nutrients.

[91] Moniaga C.S., Tominaga M., Takamori K. (2022). An Altered Skin and Gut Microbiota Are Involved in the Modulation of Itch in Atopic Dermatitis. Cells.

[92] Millard A.L., Mertes P.M., Ittelet D., Villard F., Jeannesson P., Bernard J. (2002). Butyrate Affects Differentiation, Maturation and Function of Human Monocyte-Derived Dendritic Cells and Macrophages. Clin. Exp. Immunol..

[93] Codoñer F.M., Ramírez-Bosca A., Climent E., Carrión-Gutierrez M., Guerrero M., Pérez-Orquín J.M., Horga De La Parte J., Genovés S., Ramón D., Navarro-López V. (2018). Gut Microbial Composition in Patients with Psoriasis. Sci. Rep..

[94] Samuelson D.R., Welsh D.A., Shellito J.E. (2015). Regulation of Lung Immunity and Host Defense by the Intestinal Microbiota. Front. Microbiol..

[95] Fujimura K.E., Demoor T., Rauch M., Faruqi A.A., Jang S., Johnson C.C., Boushey H.A., Zoratti E., Ownby D., Lukacs N.W. (2014). House Dust Exposure Mediates Gut Microbiome Lactobacillus Enrichment and Airway Immune Defense against Allergens and Virus Infection. Proc. Natl. Acad. Sci. USA.

[96] Trompette A., Gollwitzer E.S., Yadava K., Sichelstiel A.K., Sprenger N., Ngom-Bru C., Blanchard C., Junt T., Nicod L.P., Harris N.L. (2014). Gut Microbiota Metabolism of Dietary Fiber Influences Allergic Airway Disease and Hematopoiesis. Nat. Med..

[97] Chassaing B., Ley R.E., Gewirtz A.T. (2014). Intestinal Epithelial Cell Toll-like Receptor 5 Regulates the Intestinal Microbiota to Prevent Low-Grade Inflammation and Metabolic Syndrome in Mice. Gastroenterology.

[98] Arias Á., Vicario M., Bernardo D., Olalla J.M., Fortea M., Montalban-Arques A., Martínez-Fernández P., González-Castro A.M., Mota-Huertas T., Arias-González L. (2018). Toll-like Receptors-Mediated Pathways Activate Inflammatory Responses in the Esophageal Mucosa of Adult Eosinophilic Esophagitis. Clin. Transl. Gastroenterol..

[99] Kawai T., Akira S. (2011). Toll-like Receptors and Their Crosstalk with Other Innate Receptors in Infection and Immunity. Immunity.

[100] Zhang Y., Wang H.C., Feng C., Yan M. (2019). Analysis of the Association of Polymorphisms Rs5743708 in TLR2 and Rs4986790 in TLR4 with Atopic Dermatitis Risk. Immunol. Investig..

[101] Shi N., Li N., Duan X., Niu H. (2017). Interaction between the Gut Microbiome and Mucosal Immune System. Mil. Med Res..

[102] West C.E., Rydén P., Lundin D., Engstrand L., Tulic M.K., Prescott S.L. (2015). Gut Microbiome and Innate Immune Response Patterns in IgE-Associated Eczema. Clin. Exp. Allergy.

[103] Ellis S.R., Nguyen M., Vaughn A.R., Notay M., Burney W.A., Sandhu S., Sivamani R.K. (2019). The Skin and Gut Microbiome and Its Role in Common Dermatologic Conditions. Microorganisms.

[104] Golpour F., Abbasi-Alaei M., Babaei F., Mirzababaei M., Parvardeh S., Mohammadi G., Nassiri-Asl M. (2023). Short Chain Fatty Acids, a Possible Treatment Option for Autoimmune Diseases. Biomed. Pharmacother..

[105] Mucida D., Park Y., Kim G., Turovskaya O., Scott I., Kronenberg M., Cheroutre H. (2007). Reciprocal TH17 and Regulatory T Cell Differentiation Mediated by Retinoic Acid. Science.

[106] Atarashi K., Tanoue T., Oshima K., Suda W., Nagano Y., Nishikawa H., Fukuda S., Saito T., Narushima S., Hase K. (2013). Treg Induction by a Rationally Selected Mixture of Clostridia Strains from the Human Microbiota. Nature.

[107] Round J.L., Mazmanian S.K. (2010). Inducible Foxp3+ Regulatory T-Cell Development by a Commensal Bacterium of the Intestinal Microbiota. Proc. Natl. Acad. Sci. USA.

[108] Clarke G., Grenham S., Scully P., Fitzgerald P., Moloney R.D., Shanahan F., Dinan T.G., Cryan J.F. (2013). The Microbiome-Gut-Brain Axis during Early Life Regulates the Hippocampal Serotonergic System in a Sex-Dependent Manner. Mol. Psychiatry.

[109] Gao K., Mu C.L., Farzi A., Zhu W.Y. (2020). Tryptophan Metabolism: A Link Between the Gut Microbiota and Brain. Adv. Nutr..

[110] Yokoyama S., Hiramoto K., Koyama M., Ooi K. (2015). Impairment of Skin Barrier Function via Cholinergic Signal Transduction in a Dextran Sulphate Sodium-Induced Colitis Mouse Model. Exp. Dermatol..

[111] Cryan J.F., Dinan T.G. (2012). Mind-Altering Microorganisms: The Impact of the Gut Microbiota on Brain and Behaviour. Nat. Rev. Neurosci..

[112] Murdaca G., Greco M., Borro M., Gangemi S. (2021). Hygiene Hypothesis and Autoimmune Diseases: A Narrative Review of Clinical Evidences and Mechanisms. Autoimmun. Rev..

[113] Kıykım A., Öğülür İ., Yazıcı D., Çokuğraş H., Akdiş M., Akdiş C.A. (2023). Epithelial Barrier Hypothesis and Its Comparison with the Hygiene Hypothesis. Turk. Arch. Pediatr..

[114] Haahtela T. (2019). A Biodiversity Hypothesis. Allergy.

[115] Roth-Walter F., Afify S.M., Pacios L.F., Blokhuis B.R., Redegeld F., Regner A., Petje L.M., Fiocchi A., Untersmayr E., Dvorak Z. (2021). Cow’s Milk Protein β-Lactoglobulin Confers Resilience against Allergy by Targeting Complexed Iron into Immune Cells. J. Allergy Clin. Immunol..

[116] Loh E.D.W., Yew Y.W. (2022). Hand Hygiene and Hand Eczema: A Systematic Review and Meta-Analysis. Contact Dermat..

[117] Vindenes H.K., Drengenes C., Amin H., Irgens-Hansen K., Svanes C., Bertelsen R.J. (2024). Longitudinal Analysis of the Skin Microbiome in Association with Hand Eczema, Hand Hygiene Practices and Moisturizer Use. J. Eur. Acad. Dermatol. Venereol..

[118] Sherriff A., Golding J. (2002). Hygiene Levels in a Contemporary Population Cohort Are Associated with Wheezing and Atopic Eczema in Preschool Infants. Arch. Dis. Child..

[119] Łubiech K., Twarużek M. (2020). Lactobacillus Bacteria in Breast Milk. Nutrients.

[120] Hoskinson C., Dai D.L.Y., Del Bel K.L., Becker A.B., Moraes T.J., Mandhane P.J., Finlay B.B., Simons E., Kozyrskyj A.L., Azad M.B. (2023). Delayed Gut Microbiota Maturation in the First Year of Life Is a Hallmark of Pediatric Allergic Disease. Nat. Commun..

[121] Munblit D., Verhasselt V. (2016). Allergy Prevention by Breastfeeding: Possible Mechanisms and Evidence from Human Cohorts. Curr. Opin. Allergy Clin. Immunol..

[122] Moossavi S., Miliku K., Sepehri S., Khafipour E., Azad M.B. (2018). The Prebiotic and Probiotic Properties of Human Milk: Implications for Infant Immune Development and Pediatric Asthma. Front. Pediatr..

[123] Gołębiewski M., Łoś-Rycharska E., Sikora M., Grzybowski T., Gorzkiewicz M., Krogulska A. (2021). Mother’s Milk Microbiome Shaping Fecal and Skin Microbiota in Infants with Food Allergy and Atopic Dermatitis: A Pilot Analysis. Nutrients.

[124] Sánchez C., Fente C., Regal P., Lamas A., Lorenzo M.P. (2021). Human Milk Oligosaccharides (HMOs) and Infant Microbiota: A Scoping Review. Foods.

[125] Lee Y.H., Verma N.K., Thanabalu T. (2021). Prebiotics in Atopic Dermatitis Prevention and Management. J. Funct. Foods.

[126] Karaman S., Can D. (2023). Breastfeeding and Atopic Dermatitis. Breastfeeding and Metabolic Programming.

[127] Dotterud C.K., Storrø O., Johnsen R., Øien T. (2010). Probiotics in Pregnant Women to Prevent Allergic Disease: A Randomized, Double-Blind Trial. Br. J. Dermatol..

[128] Rajani P.S., Seppo A.E., Järvinen K.M. (2018). Immunologically Active Components in Human Milk and Development of Atopic Disease, with Emphasis on Food Allergy, in the Pediatric Population. Front. Pediatr..

[129] Oddy W.H., Rosales F. (2010). A Systematic Review of the Importance of Milk TGF-β on Immunological Outcomes in the Infant and Young Child. Pediatr. Allergy Immunol..

[130] Morita Y., Campos-Alberto E., Yamaide F., Nakano T., Ohnisi H., Kawamoto M., Kawamoto N., Matsui E., Kondo N., Kohno Y. (2018). TGF-β Concentration in Breast Milk Is Associated with the Development of Eczema in Infants. Front. Pediatr..

[131] Wegh C.A.M., Geerlings S.Y., Knol J., Roeselers G., Belzer C. (2019). Postbiotics and Their Potential Applications in Early Life Nutrition and Beyond. Int. J. Mol. Sci..

[132] Snijders B.E.P., Thijs C., Dagnelie P.C., Stelma F.F., Mommers M., Kummeling I., Penders J., van Ree R., van den Brandt P.A. (2007). Breast-Feeding Duration and Infant Atopic Manifestations, by Maternal Allergic Status, in the First 2 Years of Life (KOALA Study). J. Pediatr..

[133] Kull I., Böhme M., Wahlgren C.F., Nordvall L., Pershagen G., Wickman M. (2005). Breast-Feeding Reduces the Risk for Childhood Eczema. J. Allergy Clin. Immunol..

[134] Al-Abadie M., Beer G., Al-Rubaye M., Oumeish F., Abadie D. (2021). Does Breastfeeding Delay the Onset of Eczema in Infants?. Egypt. J. Dermatol. Venerol..

[135] Lien T.Y., Goldman R.D. (2011). Breastfeeding and Maternal Diet in Atopic Dermatitis. Can. Fam. Physician.

[136] Wang J., Ramette A., Jurca M., Goutaki M., Beardsmore C.S., Kuehni C.E. (2017). Association between Breastfeeding and Eczema during Childhood and Adolescence: A Cohort Study. PLoS ONE.

[137] Zhao H., Zhou J., Lu H., Xi A., Luo M., Wang K., Lv H., Wang H., Wang P., Miao J. (2022). Azithromycin Pretreatment Exacerbates Atopic Dermatitis in Trimellitic Anhydride-Induced Model Mice Accompanied by Correlated Changes in the Gut Microbiota and Serum Cytokines. Int. Immunopharmacol..

[138] Kuperman A.A., Koren O. (2016). Antibiotic Use during Pregnancy: How Bad Is It?. BMC Med..

[139] Miyoshi J., Hisamatsu T. (2022). The Impact of Maternal Exposure to Antibiotics on the Development of Child Gut Microbiome. Immunol. Med..

[140] Chang Y.C., Wu M.C., Wu H.J., Liao P.L., Wei J.C.C. (2023). Prenatal and Early-Life Antibiotic Exposure and the Risk of Atopic Dermatitis in Children: A Nationwide Population-Based Cohort Study. Pediatr. Allergy Immunol..

[141] Yu J. (2023). Are Perinatal Antibiotics Responsible for Atopic Dermatitis? The Debate Rages on. Br. J. Dermatol..

[142] Wan M., Yang X. (2023). Maternal Exposure to Antibiotics and Risk of Atopic Dermatitis in Childhood: A Systematic Review and Meta-Analysis. Front. Pediatr..

[143] Yamamoto-Hanada K., Yang L., Narita M., Saito H., Ohya Y. (2017). Influence of Antibiotic Use in Early Childhood on Asthma and Allergic Diseases at Age 5. Ann. Allergy Asthma Immunol..

[144] Li W., Yosipovitch G. (2020). The Role of the Microbiome and Microbiome-Derived Metabolites in Atopic Dermatitis and Non-Histaminergic Itch. Am. J. Clin. Dermatol..

[145] Saarialho-Kere U. (2004). The Gut-Skin Axis. J. Pediatr. Gastroenterol. Nutr..

[146] Kim W.K., Jang Y.J., Han D.H., Jeon K., Lee C., Han H.S., Ko G.P. (2020). Lactobacillus Paracasei KBL382 Administration Attenuates Atopic Dermatitis by Modulating Immune Response and Gut Microbiota. Gut Microbes.

[147] Kwon H.K., Lee C.G., So J.S., Chae C.S., Hwang J.S., Sahoo A., Nam J.H., Rhee J.H., Hwang K.C., Im S.H. (2010). Generation of Regulatory Dendritic Cells and CD4+Foxp3+ T Cells by Probiotics Administration Suppresses Immune Disorders. Proc. Natl. Acad. Sci. USA.

[148] Choi W.J., Konkit M., Kim Y., Kim M.K., Kim W. (2016). Oral Administration of Lactococcus Chungangensis Inhibits 2,4-Dinitrochlorobenzene-Induced Atopic-like Dermatitis in NC/Nga Mice. J. Dairy Sci..

[149] Martinez F.A.C., Balciunas E.M., Converti A., Cotter P.D., De Souza Oliveira R.P. (2013). Bacteriocin Production by *Bifidobacterium* Spp. A Review. Biotechnol. Adv..

[150] Enomoto T., Sowa M., Nishimori K., Shimazu S., Yoshida A., Yamada K., Furukawa F., Nakagawa T., Yanagisawa N., Iwabuchi N. (2014). Effects of Bifidobacterial Supplementation to Pregnant Women and Infants in the Prevention of Allergy Development in Infants and on Fecal Microbiota. Allergol. Int..

[151] Roessler A., Friedrich U., Vogelsang H., Bauer A., Kaatz M., Hipler U.C., Schmidt I., Jahreis G. (2008). The Immune System in Healthy Adults and Patients with Atopic Dermatitis Seems to Be Affected Differently by a Probiotic Intervention. Clin. Exp. Allergy.

[152] Climent E., Martinez-blanch J.F., Llobregat L., Ruzafa-costas B., Carrión-gutiérrez M.Á., Ramírez-boscá A., Prieto-merino D., Genovés S., Codoñer F.M., Ramón D. (2021). Changes in Gut Microbiota Correlates with Response to Treatment with Probiotics in Patients with Atopic Dermatitis. A Post Hoc Analysis of a Clinical Trial. Microorganisms.

